# Human biventricular electromechanical simulations on the progression of electrocardiographic and mechanical abnormalities in post-myocardial infarction

**DOI:** 10.1093/europace/euaa405

**Published:** 2021-03-04

**Authors:** Zhinuo J Wang, Alfonso Santiago, Xin Zhou, Lei Wang, Francesca Margara, Francesc Levrero-Florencio, Arka Das, Chris Kelly, Erica Dall'Armellina, Mariano Vazquez, Blanca Rodriguez

**Affiliations:** 1 Department of Computer Science, University of Oxford, Wolfson Building, Parks Road, Oxford OX1 3QD, UK; 2 Department of Computer Applications in Science and Engineering, Barcelona Supercomputing Centre (BSC), Barcelona, Spain; 3 ELEM Biotech, Barcelona, Spain; 4 Department of Biomedical Imaging Sciences, Leeds Institute of Cardiovascular and Metabolic Medicine, University of Leeds, Leeds, UK

**Keywords:** Computer modelling, Electromechanical simulations, Myocardial infarction, Electrocardiogram, Ejection fraction

## Abstract

**Aims:**

Develop, calibrate and evaluate with clinical data a human electromechanical modelling and simulation framework for multiscale, mechanistic investigations in healthy and post-myocardial infarction (MI) conditions, from ionic to clinical biomarkers.

**Methods and results:**

Human healthy and post-MI electromechanical simulations were conducted with a novel biventricular model, calibrated and evaluated with experimental and clinical data, including torso/biventricular anatomy from clinical magnetic resonance, state-of-the-art human-based membrane kinetics, excitation–contraction and active tension models, and orthotropic electromechanical coupling. Electromechanical remodelling of the infarct/ischaemic region and the border zone were simulated for ischaemic, acute, and chronic states in a fully transmural anterior infarct and a subendocardial anterior infarct. The results were compared with clinical electrocardiogram and left ventricular ejection fraction (LVEF) data at similar states. Healthy model simulations show LVEF 63%, with 11% peak systolic wall thickening, QRS duration and QT interval of 100 ms and 330 ms. LVEF in ischaemic, acute, and chronic post-MI states were 56%, 51%, and 52%, respectively. In linking the three post-MI simulations, it was apparent that elevated resting potential due to hyperkalaemia in the infarcted region led to ST-segment elevation, while a large repolarization gradient corresponded to T-wave inversion. Mechanically, the chronic stiffening of the infarct region had the benefit of improving systolic function by reducing infarct bulging at the expense of reducing diastolic function by inhibiting inflation.

**Conclusion:**

Our human-based multiscale modelling and simulation framework enables mechanistic investigations into patho-physiological electrophysiological and mechanical behaviour and can serve as testbed to guide the optimization of pharmacological and electrical therapies.

**What’s new?**

- A human-based biophysically detailed electromechanical biventricular multiscale model constructed and evaluated using experimental and clinical data.
- Simulations successfully reproduced both clinical electrocardiogram and mechanical phenotypes in healthy and for three post-myocardial infarction states.
- In the ischaemic model (hours post-occlusion), ST-segment elevation is caused by resting potential elevation due to hyperkalaemia, whereas in acute and chronic infarction, T-wave inversion arises due to large repolarization gradient across the infarct border zone.
- Stiffening of the infarct region had the benefit of improving systolic function by reducing infarct bulging at the expense of reducing diastolic function in chronic infarction.
- A sensitivity analysis quantified the dependency of ejection fraction on passive and active mechanical behaviour as well as arterial compliance and resistance.

## Introduction

Coronary heart disease is a leading cause of mortality worldwide, with 7.3 million deaths in 2001.^1^ One of its consequences is myocardial infarction (MI), caused by coronary artery occlusion or narrowing, which may result in myocardial damage, increased risk of sudden arrhythmic death, and heart failure.^2^ The electrocardiogram (ECG) is the most widely used clinical diagnostic tool for cardiac disease and MI. ST-segment elevation and T-wave inversion are markers of cardiac remodelling associated with different stages of MI. It is still partly unclear how ECG abnormalities reflect post-MI properties such as infarct size and location, and arrhythmic risk. 

Furthermore, left ventricular ejection fraction (LVEF) is one of the key metrics used for risk stratification in post-MI, and for decisions on treatment options such as defibrillator implantation.^3^ This is clearly suboptimal as a significant number of sudden deaths occur in patients with relatively preserved LVEF (36–50%) and a substantial proportion of patients with defibrillators do not make use of them. A deep mechanistic understanding of the variable substrate in post-MI, and how it reflects in clinical ECG and mechanical markers is needed to improve patients’ risk stratification and management.

A myriad of factors determine patient outcome post-MI including electrophysiological remodelling of ionic currents and calcium dynamics, in addition to structural abnormalities such as infarct scar size and fibrosis.^2^^,^^4^ The interplay between electrophysiological, mechanical, and structural abnormalities post-MI is very complex and hard to disentangle. Experimental animal models are available for mechanistic investigations but they present important limitations and differences with respect to humans.^5^ Computer simulations using multiscale models offer a powerful tool for mechanistic investigations on the patho-physiology of post-MI, with high spatio-temporal resolution and complete observability.

In this study, we present human biventricular electromechanical simulations in healthy conditions, and over the course of post-MI, using a novel, human-based multiscale modelling framework. Model development, calibration and evaluation are conducted based on a wide range of experimental and clinical datasets from ionic to ECG and whole-organ mechanical metrics such as LVEF.

## Methods

### Healthy biventricular electromechanical model

**Figure 1:**
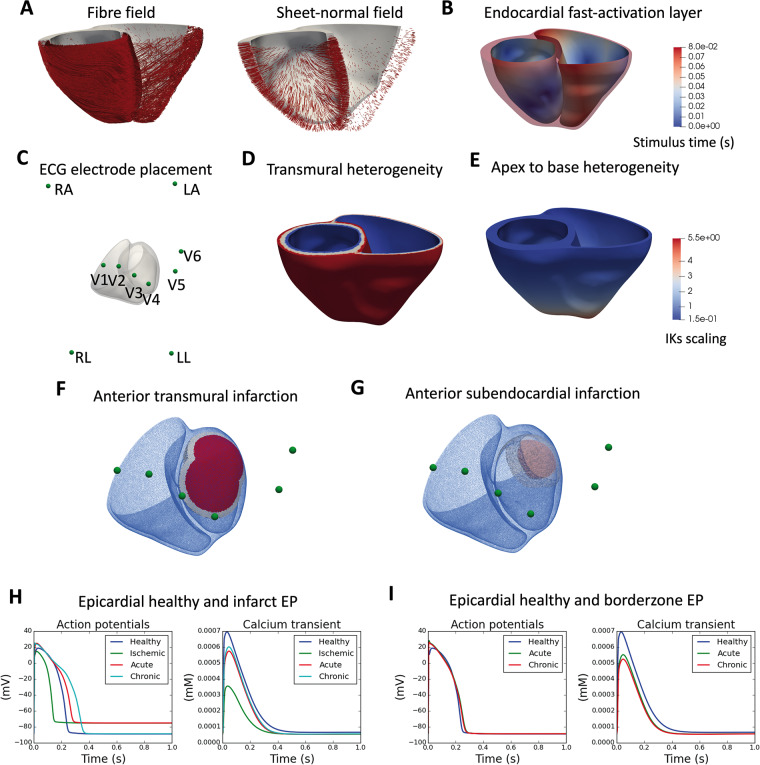
Human biventricular electromechanical model properties in healthy and post-MI conditions. (*A*) Fibre and sheet-normal field. (*B*) Colour map for local times of activation in the endocardial fast activation layer in milliseconds. (*C*) ECG electrode placement in 3D space around the biventricular mesh. (*D*) Electrophysiological transmural heterogeneity, with blue—endocardial cell type, white—mid-myocardial cell type, and red—epicardial cell type. (*E*) Electrophysiological apex-to-base heterogeneity of the slowly activating delayed rectifier potassium channel current (IKs), with colour map showing conductance for the slow rectifier potassium current. (*F*) Infarct (red), border zone (white), and remote zone (transparent blue) for an anterior endocardial infarction. (*G*) Infarct (red), border zone (white), and remote zone (transparent blue) for a transmural left anterior descending artery infarction. (*H*) Epicardial action potentials and calcium transients for the baseline, ischaemic, acute, and chronic infarct regions. (*I*) Epicardial action potentials and calcium transient for the baseline, acute, and chronic borderzone regions.

A torso-biventricular mesh (*Figure 1*) was generated from the end-diastolic magnetic resonance imaging (MRI) of a healthy subject,^6^ with the ventricular volume scaled to achieve a diastasis resting volume at roughly midway between the target end-diastolic and end-systolic volumes (*Table 1*) with an average element edge length of 220 μm. 

**Table 1:** Electrophysiological and mechanical biomarkers comparison between literature values and simulation results with healthy model

Biomarkers	Literature values	Model simulation results
Electrophysiological biomarkers		
QRS duration (ms)	96 ± 9 in men, 85 ± 6 in women (7)	100
QT interval (ms)	350–440 s (8)	330
Mechanical biomarkers		
LVEDV (mL)	142 ± 21 (SSFP-CMR) (9)	155
RVEDV (mL)	144 ± 23 (SSFP-CMR) (10)	160
LVESV (mL)	47 ± 10 (SSFP-CMR) (9)	57
RVESV (mL)	50 ± 14 (SSFP-CMR) (10)	67
LVEF (%)	67 ± 4.6 (SSFP-CMR) (9), 62 ± 7 (RNV) (11)	63
RVEF (%)	48 ± 5 (RNV) (11)	57
Peak LV pressure (mmHg)	111 ± 4 (12)	108
Peak RV pressure (mmHg)	38–40 (13)	42
Peak longitudinal fractional shortening (%)	16 ± 2%, ES mid-ventricular mid-wall (DENSE MRI) (14)	11% shortening from rest, 18% from end-diastole
Peak wall thickening (%)	33 ± 10%, radial strain, ES mid-ventricular mid-wall (DENSE MRI) (14)	36 ± 19% averaged over entire mesh from rest
Peak torsion angle (°)	peak twist 11.5 ± 3.3° (apex–base) (tagged MRI) (15)	0°

Where applicable, imaging methods are detailed in parentheses.

DENSE-MRI, displacement encoded with stimulated echoes magnetic resonance imaging; EDV, end-diastolic volume; EF, ejection fraction; ESV, end-systolic volume; LV, left ventricle; MRI, magnetic resonance imaging; RNV, radionuclide ventriculography; RV, right ventricle; SSFP-CMR, steady-state free precession cardiac magnetic resonance; SV, stroke volume.

### Human biophysically detailed electrophysiological model

Electrical propagation in the biventricular mesh was simulated using the monodomain equation with orthotropic diffusion, with cellular membrane kinetics represented by the recent ToR-ORd model^16^ (*Figure 1H*) and fibre architecture based on rule-based fields for the fibre, sheet, and inter-sheet directions^17^ (*Figure 1A*). Monodomain diffusivities were calibrated to achieve a conduction velocity of 67 cm/s, 30 cm/s, and 17 cm/s along the fibre, sheet, and sheet-normal directions.^18^ A physiological transmural and apex-to-base electrophysiological heterogeneity (*Figure 1D and E*) was applied as in ref.^6^ (details in Supplementary material online, *SM4*). Electrical stimulus via Purkinje-myocardial junctions was simulated by a fast-activation layer on the endocardial surface of the left ventricle and right ventricle with root node location to achieve realistic QRS complexes morphologies (*Figure 1B*).^19^ The simulated 12-lead ECG was computed at clinically standard lead locations in the torso (as in ref.^6^ as pseudo-ECGs) (*Figure 1C*). 

### Human mechanical model

Myocyte calcium-dependent active contractile force generation was modelled using the human-based active tension Land *et al*. model,^20^ coupled to the ToR-ORd as in ref.^21^ Strongly coupled electromechanics with orthotropic passive mechanical behaviour and balance of linear momentum with inertial effects were achieved as in ref.^22^ Active tension in the sheet direction was set to the 30% of that in the longitudinal myocyte direction, and an elastic spring boundary condition was set on the epicardial surface.

The biventricular pressure and volume behaviour was controlled using a five-phased state machine (details provided in Supplementary material online, *SM1*), which had the important addition compared to ref.^22^ of an active diastolic inflation phase that preceded electrical activation and contraction and a passive diastolic inflation phase that followed isovolumetric relaxation, where elastic recoil was allowed to occur. The basal plane was rigidly fixed in space to prevent unphysiological tilting and expansion at the base (see Supplementary material online, *SM10*). Other boundary conditions were as in ref.^22^

### Model calibration

**Figure 2:**
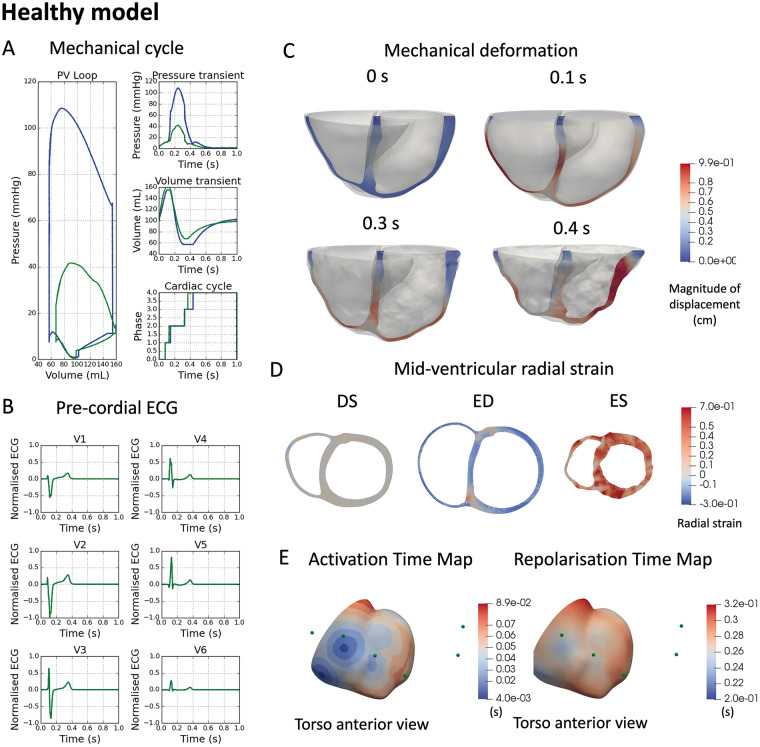
Healthy electromechanical simulations. (*A*) Pressure–volume loop for both left ventricle (blue) and right ventricle (green) (left), pressure transient (right top), volume transient (right middle), and cardiac phase change (right bottom). (*B*) Simulated ECG in pre-cordial leads. (*C*) Mechanical deformation at four time points in the cardiac cycle 0 s (initial), 0.1 s (end of diastolic filling), 0.3 s (peak pressure), 0.4 s (end of systolic ejection). (*D*) Mid-ventricular slice showing radial strain at diastasis (DS), end-diastole (ED), and end-systole (ES). (*E*) Activation and repolarisation time maps with pre-cordial ECG locations shown as green spheres.

Three beats of 1000 ms cycle length were simulated and pressure–volume as well as ECG convergence was reached after the second beat (Supplementary material online, *SM5*). The linear parameters of the passive mechanical behaviour, spring constant for the epicardial elastic spring boundary condition, the peak active tension, and the resistance of the arterial Windkessel model were calibrated to achieve physiological peak pressure and ejection fraction (EF), building on the sensitivity analysis performed in ref.^22^ and in this study (details in Supplementary material online, *SM5*). See Supplementary material online, *SM3* for a full list of calibrated parameters. The speed of propagation of the fast-activation layer was calibrated to 89.5 cm/s to achieve a QRS duration of 100 ms on the simulated ECGs (*Figure 2B*).

### Post-myocardial infarction biventricular electromechanical model 

Two infarct geometries were generated: (i) a fully transmural anterior infarct covering 20% of the left ventricular (LV) mass, with a border zone covering a further 10% (*Figure 1F*) and (ii) a sub-endocardial anterior infarct covering 2% of LV mass and 50% maximum wall depth, with a border zone covering 5% (*Figure 1G*). For each geometry, three post-MI models were calibrated to correspond with three clinically identifiable time points: ischaemic (first hours to days), acute (first days), and chronic (days to weeks). Their electrophysiological and mechanical characteristics were as follows:

#### Ischaemic

The borderzone region was considered electromechanically healthy. The infarct region had normal conduction, with 25% inhibition of the fast sodium current (INa) and the L-type calcium current (ICaL) with hyperkalaemic extracellular potassium concentration (Ko) of 8.5 mM and switching on the ATP-sensitive potassium current, and the contractile coupling was unaltered^23^ (*Figure 1H*).

#### Acute myocardial infarction

The border zone had 42.5% inhibition of INa, 30% inhibition of ICaL, and the rapid delayed rectifier potassium current (IKr), 100% reduction in the transient outward potassium current, 40% reduction in the inward rectifier potassium current, a 33% increase of the background calcium current, an increase of Ca2/calmodulin-dependent protein kinase II (CaMKII) autophosphorylation rate (+50%), and a slower calcium release kinetics induced by CaMK II phosphorylation (tau_relp +500%) (*Figure 1I*). The conduction was reduced to a third of the normal.^24^ The infarcted region had similar remodelling as the border zone, except with hyperkalaemia (Ko = 8.5 mM) as well as 62.5% inhibition of IKr (*Figure 1H*).^4^ Active tension was set to zero to represent the myocyte damage and troponin release.

#### Chronic myocardial infarction

The border zone was the same as the acute stage except for a 60% inhibition of INa and 36% inhibition of ICaL (*Figure 1I*). The infarct region was the same as at the acute stage except for a return to healthy Ko (= 5 mM) and a stronger inhibition of the INa of 60% with a 36% inhibition of the ICaL (*Figure 1H*). The linear passive stiffness parameters of the infarct region were increased 200-fold to mimic fibrotic scar formation (comparable to scaling used in ref.^25^) and to produce a significant change in LV end-diastolic volume. While this was a sharp change in passive stiffness between the scar and bordering regions, this did not cause computational problems in our simulations.

### Simulation software and computational framework

All simulations were conducted using the high-performance numerical software, Alya, for complex coupled multi-physics and multi-scale problems^26^ on the supercomputer SuperMUC-NG (Leibniz Supercomputing Centre of the Bavarian Academy of Sciences, Germany). A detailed description of the cardiac baseline electromechanical model is available in refs.^22^^,^^26^ The strong coupling of electrics and mechanics follows a multi-code strategy, and these different modules are solved implicitly to achieve a global convergence every step. Simulating three heart beats of the baseline model takes 360 cores approximately 6 h to complete.

### Comparison to clinical electrocardiogram recordings

The simulated ECGs were compared with clinical recordings of the PTB Diagnostic ECG database v1.0.0 from the Physionet repository (https://physionet.org/)^27^^,^^28^  Supplementary material online, *SM* for changes occurred from acute ischaemia to MI stages. Another set of clinical ECGs was obtained from one patient with acute anterior MI to demonstrate the long-term recovery of post-MI ECG.

### Ethics approval and consent to participate

The clinical protocol was approved by the National Research Ethics Service (17/YH/0062) in the UK. The study complied with the Declaration of Helsinki and all subjects gave written informed consent.

## Results

### Electromechanical simulations in healthy conditions

*Figure 2* illustrates simulated mechanical deformation (*A, C, D*), and electrophysiological function (*B, E*) using the healthy electromechanical model (with parameter values as in Supplementary material online, *Tables in SM3*). *Table 1* shows that clinically relevant electrophysiological and mechanical biomarkers are in range with clinical data reported in the literature (expanded version provided in Supplementary material online, *SM6*). The simulated ECG reports a physiological R wave progression, and QRS and QT intervals of 100 and 330 ms (*Table 1* and *Figure 2B*). Activation and repolarization time maps show the normal electrical propagation and repolarization sequences (*Figure 2E*). Importantly, LVEF is 63%, peak systolic pressure is 108 mmHg, with 11% systolic longitudinal fractional shortening (*Figure 2C*, 0.4 s) and an averaged 36% systolic wall thickening (*Figure 2D*), all within physiological range (references in *Table 1*). Systolic torsion angle however was not reproduced in the simulations (*Table 1*). As shown in Supplementary material online, *SM5*, sensitivity analysis reports that varying pericardium stiffness and endocardial boundary conditions can have strong effects on mechanical deformation. Although tuning these parameters was important, a significant diastolic inflation preceding electrical activation and a fixed basal plane were critical to achieve EF above 50%. Diastolic inflation affected EF via the Frank-Starling mechanisms, and the fixed basal plane prevented unphysiological expansion and tilting of the basal plane (see Supplementary material online, *SM10* for illustration) that severely affected the systolic volumes of the simulation.

### Electromechanical simulations over the course of post-myocardial infarction

**Figure 3:**
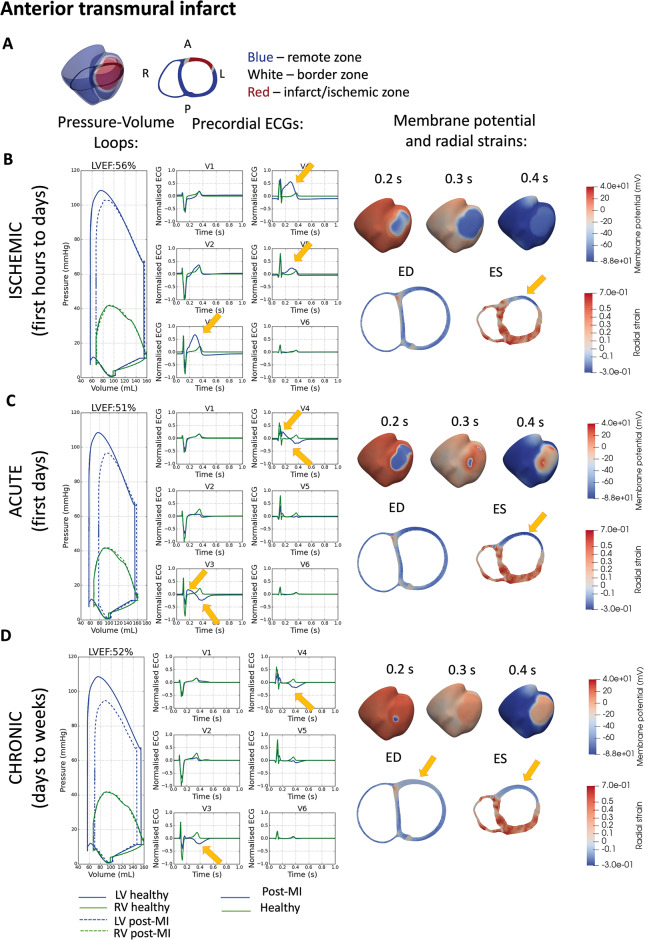
Effect of fully transmural myocardial infarction (MI) (*A*) on electromechanical function for three chronologically-ordered stages: (*B*) ischaemic, (*C*) acute post-MI, and (*D*) chronic post-MI. For each stage is shown: biventricular pressure–volume (PV) loop and pre-cordial ECG characteristics. Membrane potential plot at three consecutive time points in the cardiac cycle as labelled. Mid-ventricular short-axis slice (see *A* for slice position) showing deformation and radial strain at end-diastole (ED) and end-systole (ES). Negative strain (extension) in blue and positive strain (contraction) in red.

**Figure 4:**
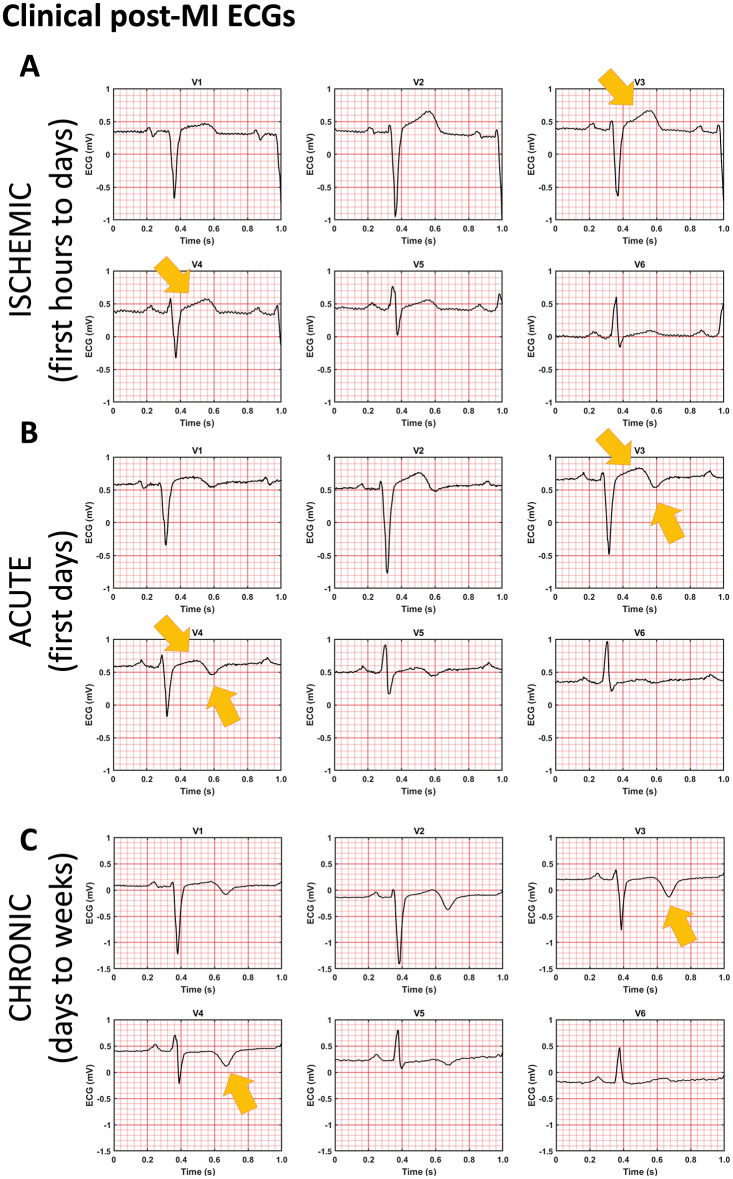
Clinical post-MI ECGs from the PTB Diagnostic ECG database (see reference in text) showing similar ST-segment and T-wave abnormalities at three chronological states post-MI as simulation. The patient had acute anterior MI on 18 October 1990 and was admitted in hospital on 19 October 1990. The catheterization date was 26 October 1990, and the three ECGs were obtained on 24 October 1990 (*A*), 29 October 1990 (*B*), and 03 December 1990 (*C*).

*Figure 3* illustrates the effect of a fully transmural anterior infarct in electromechanical function in the three stages post-MI (ischaemic, acute, and chronic). The simulated ECGs of the three post-MI stages were compared against clinical ECGs of a patient with anterior MI from the PTB Diagnostic ECG database (*Figure 4*).

In the ischaemic phase, the simulated ECG showed significant ST-segment elevation in the V3, V4, and V5 leads (*Figure 3B*), related to elevated resting potential and impaired propagation in the ischaemic region (*Figure 3B*). This is consistent with the clinical ECG in *Figure 4* obtained before catheterization therapy. Ischaemic electrophysiological abnormalities caused reduced calcium amplitude (*Figure 1H*) and active tension, which caused a 7% reduction in LVEF (*Figure 3B*). Reduction in active contraction caused systolic stretching and thinning in the infarct centre (arrow in *Figure 3B*).

In acute post-MI (*Figure 3C*), the simulated ECG showed ST-segment elevation in conjunction with T-wave inversion in V3 and V4, as shown in *Figure 4B* in the ECG 3 days after catheterization. Simulations show that the magnitude of ST-segment elevation in this phase was less severe than in the ischaemic phase (compare ECG of *Figure 3B and C*). Propagation was delayed in the infarct region (*Figure 3C*, 0.2 s), though less severely than the earlier ischaemia (compare *Figure 3B and C* at 0.3 s). At 0.4 s, there was a significant repolarization gradient which corresponded to the inverted T-wave (arrows in *Figure 3C*). In this phase, the contractile mechanism was completely turned off while scar tissue had not yet had time to form, therefore there was a large amount of infarct stretching and thinning during systole. Unsurprisingly, the LVEF suffers significantly exhibiting a 12% decrease to LVEF = 51% (*Figure 3C*). This was comparable to clinical evidence in ref.^29^ with 48 ± 8% LVEF after percutaneous coronary intervention, which was administered a median of 67.5 h after the coronary event.

In the chronic phase, the simulated ECG showed no ST-segment elevation but T-wave inversion in V3 and V4 (*Figure 3D*, ECG), as in *Figure 4C* showing the patient ECG 38 days after catheterisation. This corresponded with near-healthy propagation through the scar region combined with a high repolarisation gradient (*Figure 3D*, 0.4 s). The increase in stiffness of the infarct region due to scar formation caused a reduced radial strain at end-diastole and also reduced scar stretching during systole (*Figure 3D*, end-systole). This had the effect of reducing end-diastolic volume by 8 mL, but improving systolic function, which was reflected in the slightly improved LVEF with a reduction of 11% compared to healthy (*Figure 3D*, pressure–volume loop). This was comparable to clinical evidence in ref.^29^ of 52 ± 8% LVEF at 2 months after infarction event.

Therefore, our simulation results successfully captured the transient evolution of ECGs in different stages from acute ischaemia to MI. Although the patient in *Figure 4* showed three ECG phenotypes in a chronological order, some patients may not experience all these stages. As shown in Supplementary material online, *SM9*, patients may have ST-segment resolution immediately after percutaneous coronary intervention, and the normal T-wave polarity can be maintained (Supplementary material online, *Figure SM9*).

Supplementary material online, *Figure SM7* shows simulation results for the subendocardial anterior infarct in the three post-MI phases, following the layout in *Figure 3*. In all cases, the effect on the ECG were mild, with the acute and chronic phases showing some T-wave amplitude reduction in the V5 lead (Supplementary material online, *Figure SM7A*), which corresponded with an increased repolarization gradient on the epicardial surface (Supplementary material online, *Figure SM7B*, 0.4 s). The mechanical effect of the endocardial infarct was also very small, with reductions of 1%, 2%, and 3% on the ejection fraction for the ischaemic, acute, and chronic phases, respectively (Supplementary material online, *Figure SM7A*). The small size and more endocardial location of this infarct caused negligible regional diastolic or systolic abnormalities (Supplementary material online, *Figure SM7C*) for all three points in the chronological progression.

## Discussion

In this study, we present the development and evaluation of a multiscale modelling and simulation framework for human ventricular electromechanical function from ionic to clinical ECG and imaging biomarkers. Simulations in healthy conditions and several stages post-MI were demonstrated yielding ECG and mechanical properties within clinical data range. The multiscale nature of the human biventricular model allowed the identification of the key ionic and tissue factors explaining the evolution of ECG and mechanical abnormalities over the course of post-MI. This work opens new avenues for multiscale investigations on the signature of pathophysiological abnormalities on clinical biomarkers, arrhythmia and heart failure mechanisms and therapy development and optimization.

In healthy conditions, the simulated ECG presented realistic QRS complex morphology, R-wave progression, T-wave morphology, as well as QRS width and QT interval duration. This was achieved following previous work on root node location and endocardial speed for QRS characteristics,^6^^,^^19^ as well as membrane properties,^16^ and ionic current heterogeneity for T-wave properties.

To achieve consistency with clinical data for healthy pressure–volume characteristics as well as healthy levels of systolic longitudinal fraction shortening and wall thickening, the simulation of pressure boundary conditions in the two diastolic phases was critical. Many previous electromechanical modelling studies had a simplistic single-phased approach to diastolic filling^22^^,^^30^ where the end-diastolic state was considered the undeformed or pre-stressed state and electrophysiological stimulus was applied on a non-inflated geometry. However, physiologically, the stretching of myocardium prior to contraction is critical for healthy systolic function via the length-dependence of tension.^20^ Furthermore, the inclusion of a diastolic filling phase enabled us to explore the effect of stiff scar formation on diastolic function (*Figure 3D*). Our new formation of the passive inflation phase, which occurs at the end of the cardiac cycle and is driven by elastic recoil, was also better able to return the model to the initial state compared with.^22^

Also important to achieving healthy mechanical function was fixing the basal plane to prevent basal dilation and non-rigidity. Physiologically, a fibrous cardiac skeleton maintains the diameter of the basal, while the base moves longitudinally as a result of both atrial and ventricular forces. It was not possible to replicate the longitudinal basal motion realistically in an open-top ventricle-only mechanics model, and so we removed any basal long-axis motion in order to avoid spurious effects.

Simulations successfully recapitulate and explain the progression of ECG characteristics and mechanical abnormalities in post-MI. The progression of ECG characteristics from ischaemic to acute to chronic was achieved by embedding the infarct and border zone electrophysiological abnormalities that were known in literature in the fully coupled electromechanical whole organ model. The decreasing degree of ST-segment elevation was explained by reduced impairment of propagation and elevated resting potential in the infarct due to a combination of increasingly recovered fast sodium and L-type calcium channel conductances and a recovery from hyperkalaemia (by the chronic state). The increasing prominence of T-wave inversion over the course of post-MI was explained by an increasing repolarisation gradient due to increasing inhibition of the rapid rectifier potassium current. It was interesting that in order to T-wave inversion to develop, the infarct requires some level of recovery of electrical propagation, which explains the more chronic development of this characteristic. Future explorations of the arrhythmic risks of the post-MI states investigated in this study would be better informed by this knowledge.

Mechanically, our simulations demonstrated a progressive worsening of pumping function initially, with some recovery of LVEF after scar formation. It was interesting to note that simulations showed three different mechanisms underpinning changes in pumping function at the three post-MI states: the ischaemic mechanical dysfunction was due purely to electrophysiological dysfunction, the acute mechanical dysfunction was due to cross-bridge cycling failure, and the minor recovery of chronic mechanical function was due to structural changes in scar formation. The electrophysiological and multiscale nature of the ischaemic mechanical abnormality highlights the potential of this electromechanical model for pharmacological development and testing. The interplay between mechanical stretch and electrophysiological abnormality, especially in the acute state where the infarct region experiences the highest systolic stretch, could likely play a significant role in the high risk of arrhythmic events in this time bracket. In the chronic state, our model demonstrated the protective role of scar formation, with some recovery of systolic function at the expense of diastolic function. Future studies on the long-term effects of scar formation on remodelling of the remote regions can also be explored using this electromechanically-coupled model, where remote zone remodelling could be signalled by mechanical and/or electrophysiological queues.

We also demonstrated in this study that a subendocardial infarct caused very little disturbance to either the ECG or the pressure–volume characteristics at all three stages post-MI in line with results by Martinez-Navarro *et al*.^23^ in acute regional ischaemia. This highlights the limitations of the ECG characteristics and the LVEF to reflect the full picture of disease condition. Explorations of the arrhythmic risk of these cases could shed light on the reasons for why the LVEF is suboptimal as a stratification biomarker.

Our modelling and simulation framework demonstrated the ability to link both electrophysiological and mechanical mechanisms for post-MI pathology and paves the way for future studies that can further probe the interplay between the two. It can be used for assessing the arrhythmic risk of different size and shapes of infarction at different chronological stages post-infarction and can also act as a testbed for pharmacological development.

### Limitations

This study is subject to limitations in both the electrophysiology and mechanical aspects. We did not investigate the role of stretch activated ion channels on the electromechanical coupling of the model, which could play a role, especially in arrhythmogenesis in acute post-MI, where the infarcted tissue experiences abnormally high strains during systole bulging. Our model was also unable to generate a sufficiently large torsion to match clinically measured values. This could be due to the basal plane boundary condition, which was necessary due to the clinical MRI-based segmented geometry to prevent unphysiological motion at the truncated basal plane (see Supplementary material online, *SM10*). The future inclusion of a geometry with valvular plane would remove the need for a fixed basal plane and could help to improve the torsional behaviour. Furthermore, an investigation of the stiffness of the pericardial constraint could also improve torsional behaviour. Some deformation oscillations were observed in the systolic phase, which could be due to volumetric locking effects or several other causes and deserve further investigation. However, as the oscillations are of a mechanical nature, these effects are unlikely to affect the main conclusions of this study regarding the electrophysiological mechanisms of ECG abnormalities post-MI and the ameliorating effect of chronic scar stiffening on systolic function.

## Conclusions

Our human-based multiscale modelling and simulation framework enables mechanistic investigations into patho-physiological electrophysiological and mechanical behaviour, and can serve as a testbed to guide the optimisation of pharmacological and electrical therapies.

## Supplementary material

Supplementary material is available at *Europace* online.

## Supplementary Material

euaa405_Supplementary_Data
