# Supplementary material for: Human biventricular electromechanical simulations on the progression of electrocardiographic and mechanical abnormalities in post-myocardial infarction
Source: Europace. 2021 Mar 4;23(Suppl 1):i143–52. doi: 10.1093/europace/euaa405 (PMC7943362; doi:10.1093/europace/euaa405)
Supplement: euaa405_Supplementary_Data [file euaa405_supplementary_data.docx]

SUPPLEMENTARY MATERIAL.

## SM1. Mechanical boundary conditions

The intra-ventricular pressure (P) and volume (V) behaviour of the biventricular model was controlled using a five-phased state machine with a one-off initialisation phase as described below, where the phase transitions were triggered independently for each ventricle:

1. *Initialisation*. Both ventricles were firstly inflated to a uniform endocardial pressure of 0.5 kPa (3.75 mmHg) to reach a loaded resting endocardial volume, $V_{rest}$.
2. *Active inflation.* The pressure in both ventricular chambers is linearly increased to an end diastolic pressure of 1.5 kPa (11.3 mmHg) over t_diastole_ = 80 ms. This phase mimics the atrial contraction phase of diastolic filling and it is considered the first phase in the cardiac cycle because it follows directly from sinoatrial stimulus.
3. *Isovolumetric contraction.* Endocardial activation marks the beginning of this phase where stimulated myocytes begin generating active tension and the endocardial pressure (P) is allowed to increase such that the chamber volume (V) is kept approximately constant through the use of penalty terms:
   $dP=\frac{-1}{C_{p}}dV-\frac{1}{C_{v}}\frac{dV}{dt},$where $C_{p}$ and $C_{v}$ are the penalty terms for volume difference and volume rate, respectively. Furthermore, while $C_{v}$ is a user defined constant, $C_{p}$ is defined as:

$$C_{p}=\frac{P}{V}$$

1. *Ejection.* This phase is triggered when the ventricular pressure exceeds the arterial pressure, ${P_{art}}_{0}$, which was set to be 9 kPa (68 mmHg) in the LV and 3 kPa (22 mmHg) in the RV. A two-element Windkessel model is used to model the blood pressure of both the systemic and pulmonary circulation systems during ejection:
   $C\frac{dP_{art}}{dt}+\frac{P_{art}}{R}=\frac{-dV}{dt},$where C and R are the compliance and impedance of the circulation systems.
2. *Isovolumetric relaxation.* This phase is triggered by the reversal of ventricular volume change, i.e. $\frac{dV}{dt}>0$. Symmetrically with phase 2), here the pressure is allowed to decrease while the volume is kept constant.
3. *Passive filling.* During this phase the myocyte active tension is allowed to return to resting state and the volume is allowed to return to the initialised value $V_{rest}$ through:

$$dP=\frac{-1}{C_{p}}\left( V-V_{rest} \right)-\frac{1}{C_{v}}\frac{dV}{dt},$$

where ventricular volumes were calculated at each time step using the divergence theorem as detailed in (1). Here, both the $C_{p}$ and $C_{v}$ parameters are user defined and have been selected to allow full recovery of the initialised volume before the end of the cycle length of one second.

An elastic spring boundary condition was applied to the epicardial surface with stiffness $K_{epi}$.

## SM2. Passive and active mechanical model

The passive mechanical behaviour in the model is described using the following nearly incompressible form of the orthotropic Holzapfel-Ogden constitutive equation for the strain energy density function where the strain invariants (I1, I4f, I4s, I8fs) were evaluated after volumetric decomposition of the deformation gradient tensor:

$$\psi=\frac{K}{2}\left( J-1 \right)^{2}+\frac{a}{2b}\left( e^{b\left( I_{1}-3 \right)}-1 \right)+\sum_{i=f,s} \frac{a_{i}}{2b_{i}}\left( e^{b_{i}\left( I_{4i}-1 \right)^{2}}-1 \right)+\frac{a_{fs}}{2b_{fs}}\left( e^{b_{fs}I_{8fs}^{2}}-1 \right)$$

The active tension generation along the myocyte direction follows the formulation presented in (1,2) and a scaling parameter $T_{scale}$, which is multiplied to the maximum active tension parameter $T_{ref}$ in [1], is calibrated to achieve physiological ejection fraction. Active tension in the sheet direction was set to 30% of the active tension in the fibre direction. This modelling decision was based on (3) which demonstrated that transverse active tension was necessary in order to achieve more realistic systolic shear strain. There is also some experimental basis for this phenomenon from (4). A sensitivity analysis of this transverse activation percentage can be found in (1).

## SM3. Parameter values in the healthy mechanical model

The parameters to reproduce the healthy baseline model are presented in Tables SM3.1 and SM3.2.  The units for pressure/stiffness was Barye (Ba), which is consistent with the centimetre-gram-second system of units used in the simulations. This unit can be converted to kilopascals (kPa) via the ratio 1:0.0001 (Ba:kPa).

Table SM3.1: parameters for electrical and mechanical properties

| **Name** | **Parameter** | **Value** | **Unit** |
| --- | --- | --- | --- |
| diffusivity in fibre, sheet and sheet normal directions | d_f_ | 0.003025 | cm/mS |
|  | d_s_ | 0.000513 | cm/mS |
|  | d_n_ | 0.000283 | cm/mS |
| active mechanics: scaling parameter for active tension | T_scale_ | 12 |  |
| bulk modulus | K | 12185000 | Ba |
| passive mechanics: exponential term in isotropic matrix, fibre, sheet and normal direction | a | 200 | Ba |
|  | b | 9.242 |  |
|  | a_f_ | 300 | Ba |
|  | b_f_ | 15.972 |  |
|  | a_s_ | 200 | Ba |
|  | b_s_ | 10.446 |  |
|  | a_fs_ | 100 | Ba |
|  | b_fs_ | 11.602 |  |

Table SM3.2: parameters for boundary conditions and phase control

| **Name** | **Parameter** | **LV** | **RV** | **Unit** |
| --- | --- | --- | --- | --- |
| Pericardial stiffness | K_epi_ | 10000 | | Ba cm^-1^ |
| Time to initial pressure | **t**_0_ | 0.02 | 0.02 | s |
| Initial pressure | P_0_ | 5000 | 5000 | Ba |
| Duration of passive diastolic filling | t_diastole_ | 0.08 | 0.08 | s |
| Pressure at end of diastole | P_endd_ | 15000 | 15000 | Ba |
| Arterial compliance | C | 0.00055908 | 0.00055908 | cm^3^ Ba^−1^ |
| Arterial resistance | R | 250 | 100 | Ba s cm^−3^ |
| Aortic pressure | P_art0_ | 90000 | 20000 | Ba |
| Pressure at end of isovolumetric relaxation | P_post_ | 10000 | 10000 | Ba |
| Penalty parameters for isovolumetric contraction | C_v_ | 1 | 1 | cm^3^ s^-1^ Ba^-1^ |
| Penalty parameters for isovolumetric relaxation | C_v_ | 0.2 | 0.2 | cm^3^ s^-1^ Ba^-1^ |
| Penalty parameters for passive filling | C_p_, C_v_ | 0.2,1 | 0.2,1 | cm^3^ Ba^-1^, cm^3^ s^-1^ Ba^-1^ |

## SM4. Electrophysiological heterogeneity in the healthy model

Tissue electrophysiological heterogeneities were incorporated in our biventricular heart models as in (5). Transmural, apex to base, and interventricular cell electrophysiological heterogeneities based on experimental and clinical data from (6–9) were incorporated. Apex-to-base heterogeneities were modelled by including a gradual increase of IKs conductance from base to apex resulting in APD differences of 40 ms. Transmural heterogeneities were modelled using layers of endocardial (40% of the transmural width), mid-myocardial (30%) and epicardial cells (30%) with different AP properties as in (10). These gradients resulted in interventricular APD differences of 25 ms between left and right ventricles. The monodomain equation was used to simulate electrical propagation in the myocardium with the pseudo-ECG formation from (11,12). The pseudo-ECG approach has the advantage of reducing computational cost and has been shown to provide clinically comparable ECG recordings in (5). Furthermore, several studies suggest minimal differences in the resulting body surface potentials when assuming homogeneous or inhomogeneous torso models (13).

## SM5. Sensitivity analysis

As far as possible, the authors have aimed to use state-of-the-art electrophysiological and mechanical models and three-dimensional heterogeneities that were constructed based on human experimental data at the cellular and organ levels (2,14). It was important to investigate the uncertainties in the parameters used to characterise a model, both due to inherent variations extant in the population and also due to coupling effects of various parameters. Due to the brevity of the paper, we rely on the validation and sensitivity analyses performed in previous publications, and also the investigation into the sensitivity of the model to the mechanical parameters of this model here. We would like to point to a previous study (1) for a more in-depth analysis of the sensitivity and coupling of the parameters. Building on results from (1,5,15), a sensitivity analysis was performed to understand the variation in PV loops and ECG biomarkers to changes in key mechanical model parameters, including pericardial stiffness (K_epi_), the compliance (C) and impedance (R) of the circulation systems, active tension scaling parameters (T_scale_) and the linear passive mechanical parameters (K, a, a_f_, a_s_, a_fs_). Through high performance computing simulations, we demonstrate that a large variations (5%--40000%) in these parameters and their implication on the electromechanical response. The largest range of variation is selected to include all uncertainty in model parameters in heathy and diseased conditions. The variation in both individual and combined parameters are considered. Variations in these model parameters influence mechanical function rather than the electrophysiology and the ECG.

The variation in the pericardial stiffness is specified by scaling K_epi_. As the PV loops in Figure SM5.1 shows, the LV has smaller EDV with increasing pericardial stiffness, resulting in decreasing stroke volume and ejection fraction (EF) due to length-dependent active tension generation.

In contrast to the epicardial surface, the variation in the boundary condition on the endocardial surface (through the key parameters in the Windkessel model) affects shape of PV loops and the peak systolic pressure but does not affect the end diastolic volume (EDV). Figures SM5.2 and SM5.3 show the results obtained by varying total arterial compliance, C, and total arterial resistance, R. Similar to the observation in [1], C and R have opposite effects: the peak systolic pressure increases with an increase in R (Figure SM5.3) but decreases with an increase in C (Figure SM5.2). The EF is not sensitive to C but decreases with an increase in R.

Figure SM5.4 shows the effect of changing the scaling parameter T_scale_ , which is the scaling factor of the maximum active tension parameter T_ref_ in [1]. That is, more active tension is generated with a larger T_scale_ for the same intracellular calcium transient. As concluded in [1], increasing T_scale_ is a simple and effective method to improve the EF. Similar to this observation, increasing T_scale_ leads to a decrease in the ESV and a corresponding increase in the EF in Figure SM5.4. Figures SM5.5 and SM5.6 show the effect of scaling the linear passive mechanical parameters. In Figure SM5.5, the scaling factors are applied on all linear passive mechanical parameters (K, a, a_f_, a_s_, a_fs_). The EDV decreases as the scaling factor increases, i.e. stiffer myocardium and the EF decreases due to length-dependence of active tension generation. In order to find the parameters that have the biggest contribution, each parameter was then scaled individually. The EF decreases the fastest with an increase in bulk modulus, K, and matrix stiffness, a, but the EF was not sensitive to the other parameters (Figure SM5.6). Notably, variation in the fibre-sheet shear stiffness, a_fs_ does not affect the EF. However, the bulk modulus needs to be a large value for the incompressibility of the myocardium. Therefore, decrease in matrix stiffness, a, can effectively increase the EF.

In conclusion, the effective methods of increasing the EF include decrease in pericardial stiffness (K_epi_), total arterial resistance (R), matrix stiffness (a), and increase in active tension parameter (Tscale).

Figure SM5.1: Effects of variation in the pericardial stiffness (K_epi_) on PV loops and the LVEF. The legend in the left panel refers to the scale factors multiplying K_epi_ and the right panel shows their effect on LVEF.

Figure SM5.2: Effect of variation in the total arterial compliance, C, on PV loops and the LVEF. The numbers in legend are the scale factors multiplying C (left panel) and the right panel shows heir effect on LVEF.

Figure SM5.3: Effect of variation in the total peripheral resistance, R, on PV loops and the LVEF. The numbers in legend are the scale factors multiplied on R that are used for showing the LVEF on the right.

Figure SM5.4: Effect of variation in the active stress scaling parameter, T_scale_ , on PV loops and the LVEF. The numbers in legend are the scale factors multiplied on T_scale_ that are used to show the effect on LVEF on the right.

Figure SM5.5: Effect of scaling all linear passive mechanical parameters (K, a, af, as, afs) with same factor: the PV loops and the LVEF. The numbers in legend are the scale factors multiplied on (K, a, af, as, afs), and are used for showing the LVEF on the right.

Figure SM5.6: The EF when scaling linear passive mechanical parameters individually: each of (K, a, af, as, afs) is individually multiplied by scale factors (0.1, 0.5, 10, 50, 100, 200, 400) and the colour shows the LVEF

## SM6. Additional validation criteria for healthy baseline model

Table SM6: Electrophysiological and mechanical biomarkers comparison between literature values and baseline simulation results. Where applicable, imaging methods are detailed in parentheses. LV - left ventricle, RV - right ventricle, EDV - end diastolic volume, ESV - end systolic volume, SV - stroke volume, EF - ejection fraction. SSFP-CMR – steady-state free precession cardiac magnetic resonance, MRI: magnetic resonance imaging, RNV: radionuclide ventriculography. DENSE-MRI: displacement encoded with stimulated echoes magnetic resonance imaging.

| **Biomarkers** | **Literature values** | **Model simulation results** |
| --- | --- | --- |
| *Electrophysiological biomarkers* | | |
| QRS duration (ms) | 96 ± 9 in men, 85 ± 6 in women (16) | 100 |
| QT interval (ms) | 350 to 440 s (17) | 330 |
| *Mechanical biomarkers* | | |
| LVEDV (mL) | 120 (18),  142 ± 21 (SSFP-CMR) (19),  131 ± 24.5 (tagged MRI) (20) | 153 |
| RVEDV (mL) | 144 ± 23 (SSFP-CMR) (21) | 157 |
| LVESV (mL) | 50 (18),  47± 10 (SSFP-CMR) (19),  47.8 ± 12.0 (tagged-MRI) (20) | 57 |
| RVESV (mL) | 50 ± 14 (SSFP-CMR) (21) | 67 |
| LVSV (mL) | 70 (18),  95 ± 14 (SSFP-CMR) (20) | 96 |
| RVSV (mL) | 94 ± 15 (SSFP-CMR) (21) | 90 |
| LVEF (%) | 58 (18), 67 ± 4.6 (SSFP-CMR) (19), 63.1 ± 5.6 (CMR) (22),  62 + 7 (RNV) (23) | 63 |
| RVEF (%) | 48 + 5 (RNV) (23) | 57 |
| Peak LV pressure (mmHg) | 120 (18) | 108 |
| Peak RV pressure (mmHg) | 38-40 (24) | 42 |
| Peak longitudinal fractional shortening (%) | 16 ± 2 %, ES mid-ventricular mid-wall (DENSE MRI) (25,26) | 11 % shortening from rest, 18 % from end diastole. |
| Peak wall thickening (%) | 33 ± 10 %, radial strain, ES mid-ventricular mid-wall (DENSE MRI) (25,26) | 36 ± 19 % averaged over entire mesh from rest. |
| Peak torsion angle ($^{\circ}$) | Basal rotation -3.9 ± 1.3 degrees (clockwise), apex 7.5 ± 3.6 degrees, (anticlockwise) peak twist 11.5 ± 3.3 degrees (apex - base) (tagged MRI) (20,26) | 0$^{\circ}$ |

## SM7. Subendocardial anterior infarct results

Figure SM7 shows simulation results for the subendocardial anterior infarct in the 3 post-MI phases, following the layout in Figure 3. In all cases, the effect on the ECG were mild, with the acute and chronic phases showing some T-wave amplitude reduction in the V5 lead (Figure SM7A), which corresponded with an increased repolarisation gradient on the epicardial surface (Figure SM7B, 0.4 s). The mechanical effect of the endocardial infarct was also very small, with reductions of 1%, 2%, and 3% on the ejection fraction for the ischemic, acute, and chronic phases, respectively (Figure SM7A). The small size and more endocardial location of this infarct caused negligible regional diastolic or systolic abnormalities (Figure SM7C) for all three points in the chronological progression.


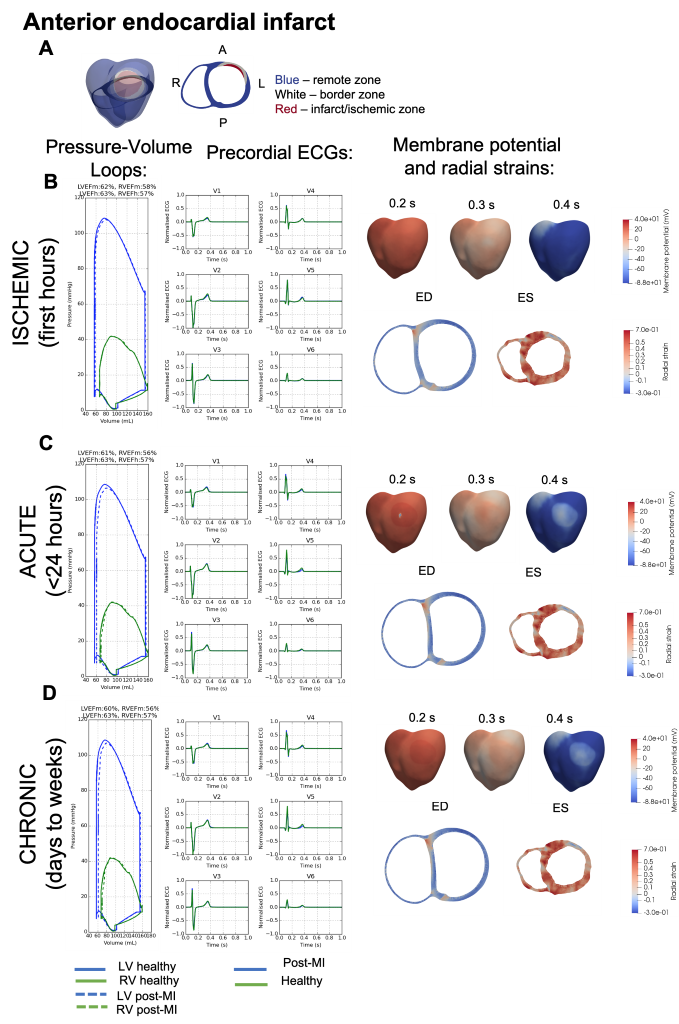


Figure SM7: Electromechanical simulation of endocardial myocardial infarction with infarct and border zone geometry shown in A), at three chronologically-ordered stages: B) ischemic, C) acute, and D) chronic. For each stage is shown: biventricular pressure volume (PV) loop and pre-cordial ECG characteristics. Membrane potential plot at three consecutive time points in the cardiac cycle as labelled. Mid-ventricular short axis slice (see A for slice position) showing deformation and radial strain at end diastole (ED) and end systole (ES). Negative strain (extension) in blue and positive strain (contraction) in red.

## SM8. Multi-beat simulation results

The mechanical and ECG characteristics of the biventricular model converged after the second cardiac cycle. Figure SM8.1, SM8.2, SM8.3, and SM8.4 demonstrates the ability of the model to return to resting state at the end of each beat and the convergence in behaviour for each of the healthy, ischemic, acute, and chronic simulations with the transmural anterior infarct.


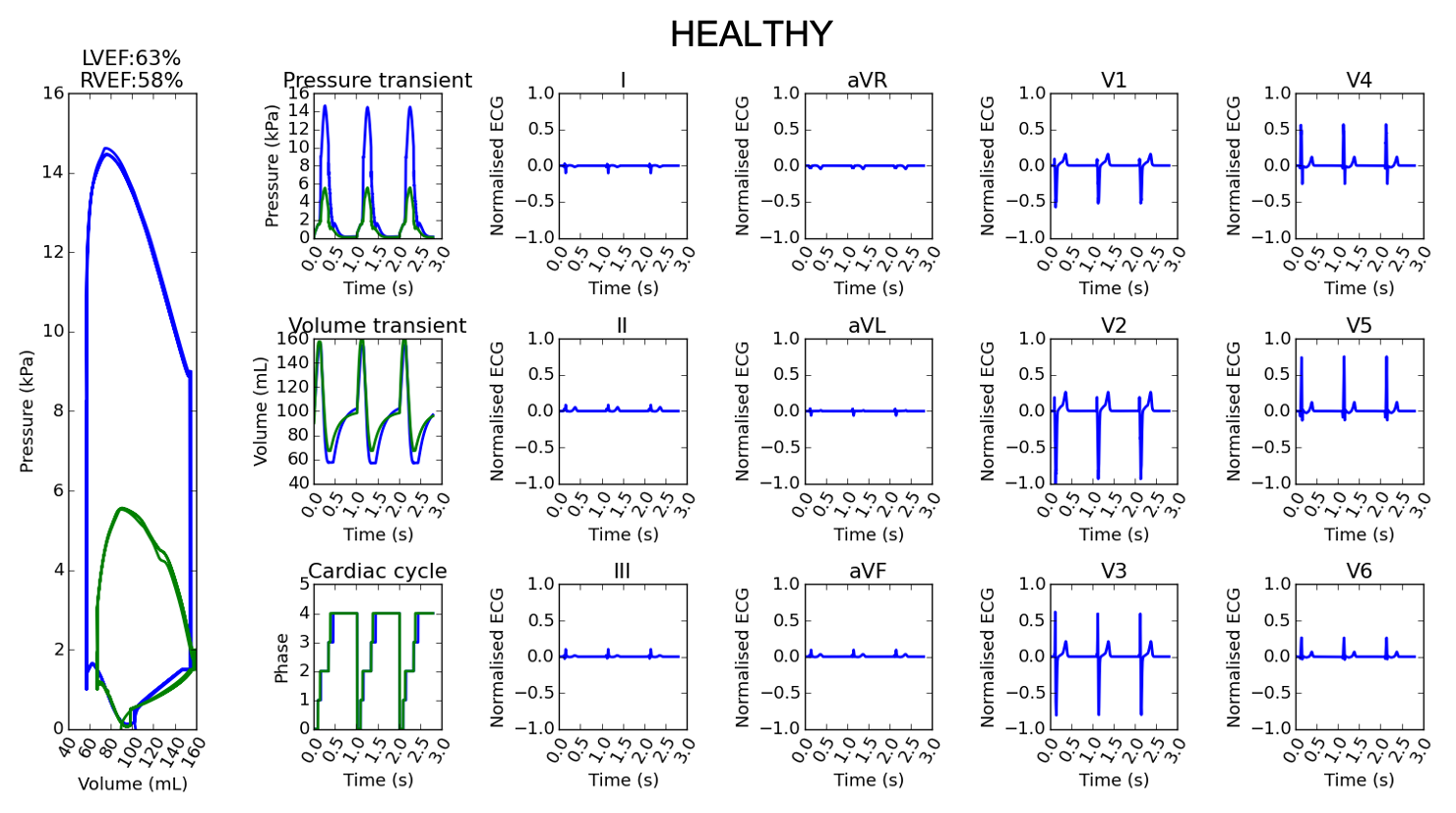


Figure SM8.1: Three beats for the healthy simulation showing pressure volume loop (left), pressure transient, volume transient, cardiac cycle phase transitions, and the full ECG (I, II, III, aVR, aVL, aVF, V1, V2, V3, V4, V5, V6).


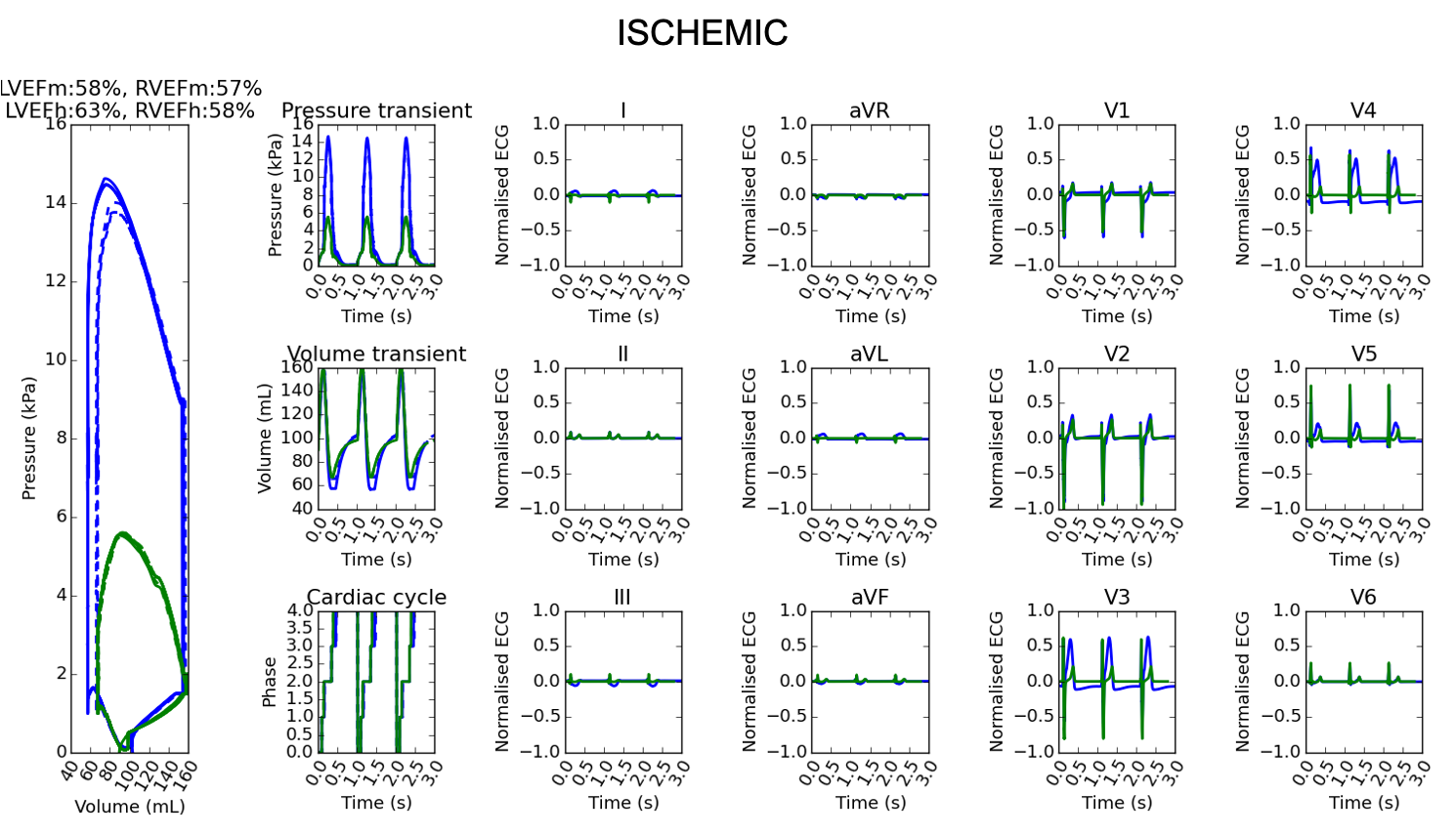


Figure SM8.2: Three beats for the ischemic simulation showing pressure volume loop (left), pressure transient, volume transient, cardiac cycle phase transitions, and the full ECG (I, II, III, aVR, aVL, aVF, V1, V2, V3, V4, V5, V6).


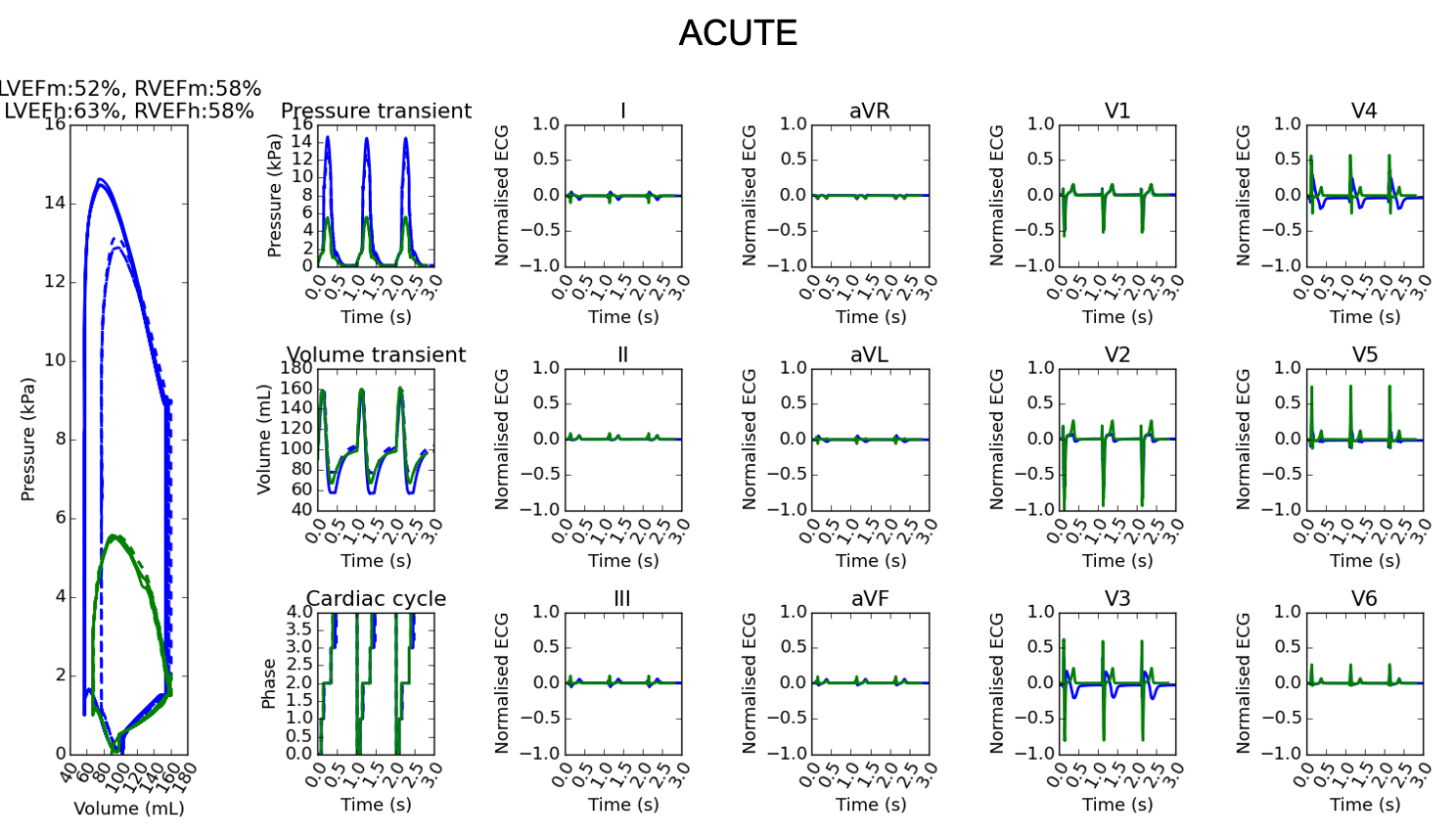


Figure SM8.3: Three beats for the acute post-MI simulation showing pressure volume loop (left), pressure transient, volume transient, cardiac cycle phase transitions, and the full ECG (I, II, III, aVR, aVL, aVF, V1, V2, V3, V4, V5, V6).


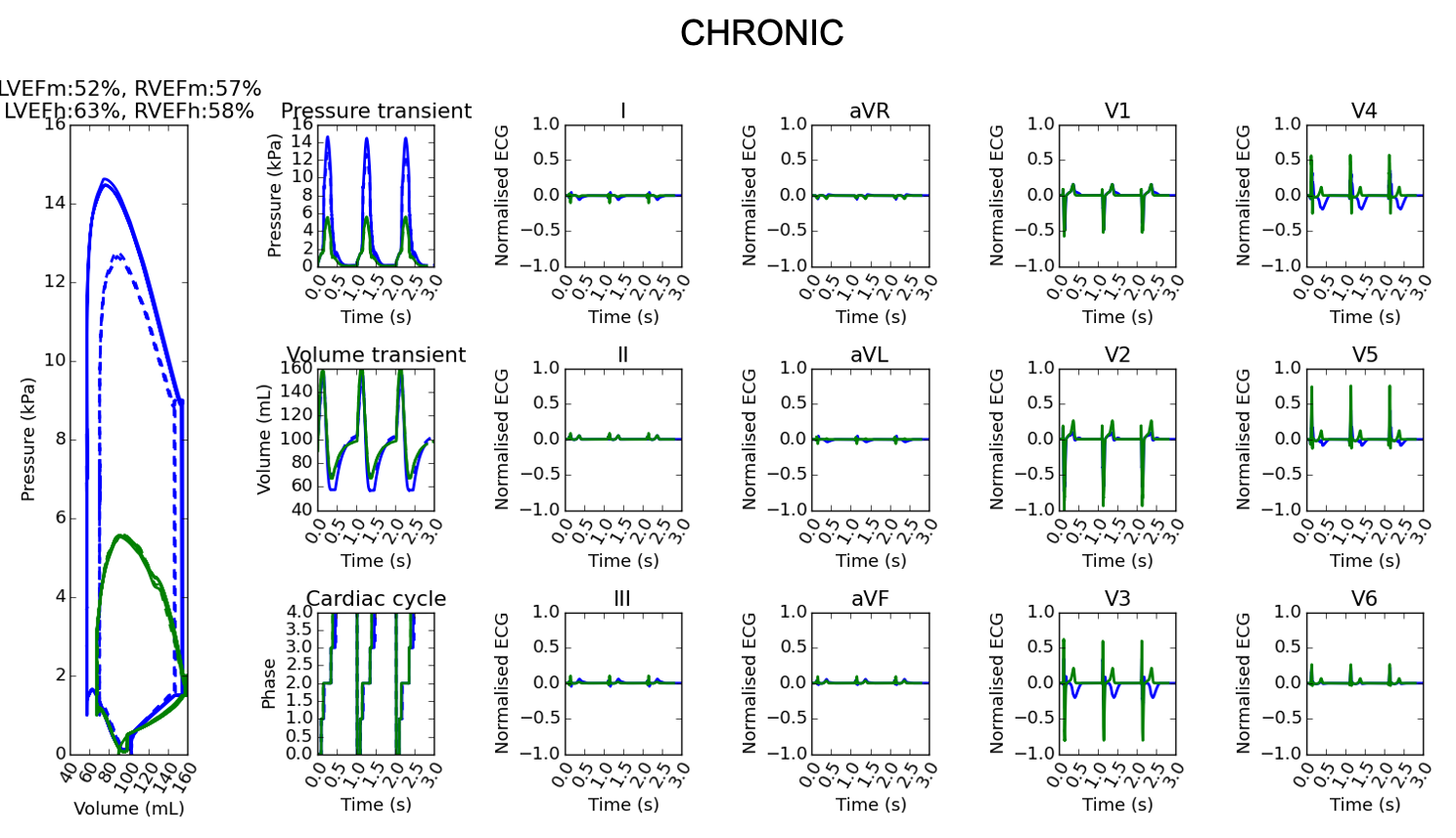


Figure SM8.4: Three beats for the chronic post-MI simulation showing pressure volume loop (left), pressure transient, volume transient, cardiac cycle phase transitions, and the full ECG (I, II, III, aVR, aVL, aVF, V1, V2, V3, V4, V5, V6).

## SM9. Clinical ECG data for recovered post-MI subjects

Clinical ECG in main text (Figure 4) was extracted from the PTB Diagnostic ECG database v1.0.0 from the Physionet repository (27,28). The clinical ECG of one patient with acute anterior MI was selected to demonstrate the recovery of post-MI electrophysiology before and after percutaneous coronary intervention (PCI).


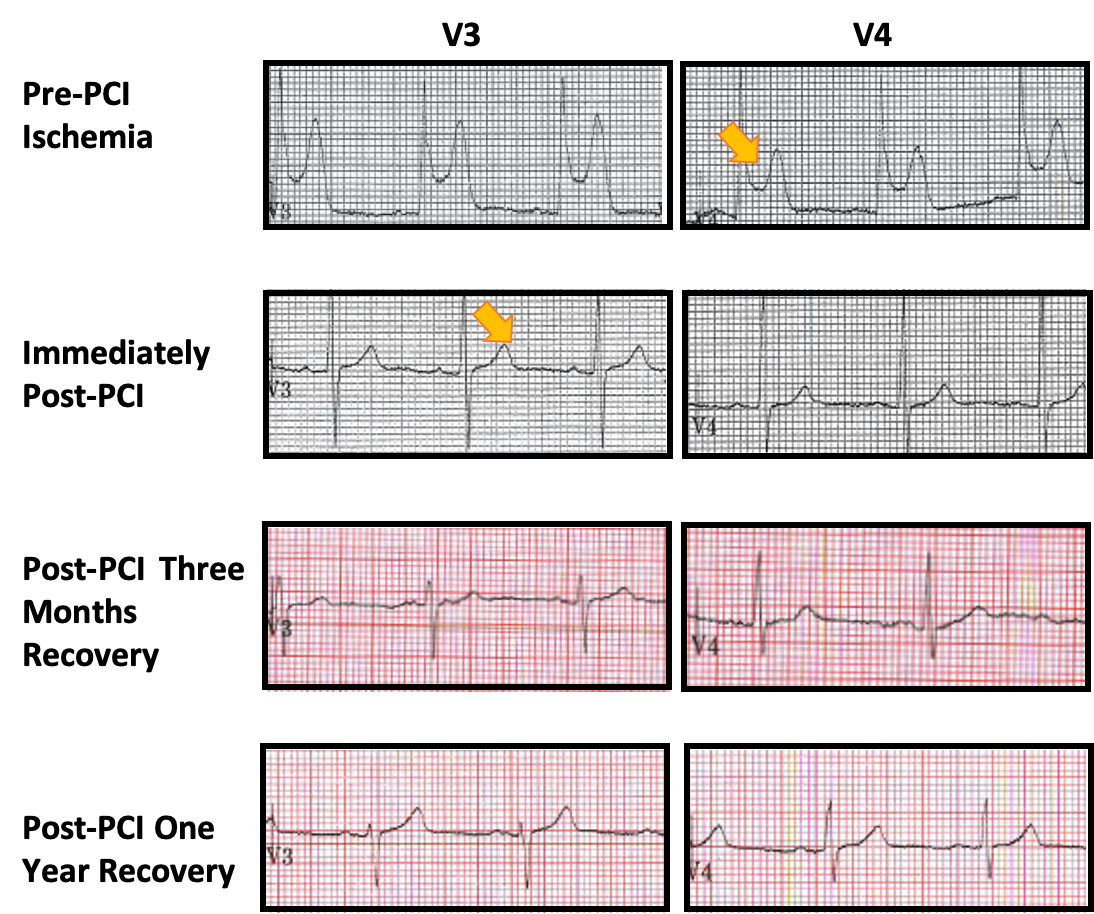


Figure SM9: Clinical ECGs of V3 and V4 leads of a patient with acute anterior ST-elevation MI. Four ECGs were obtained at different stages: 1) before PCI; 2) immediately post-PCI (taken within an hour); 3) three months post PCI; 4) one year post PCI.

## SM10. Basal boundary conditions investigation

Due to the lack of fully segmented basal anatomy, we investigated various boundary conditions at the truncated basal plane. Figure 10 shows the effect of the boundary conditions that were tried. Having no basal constraints at all causes the basal surface to no longer be planar during systole and the radius of the LV and RV opening increases dramatically during systole, causing unphysiological motion and adversely affects ejection fraction. Constraining the circumferential and radial displacement of the base prevented the increase in radius of the opening but was unable to prevent unphysiological longitudinal motion artefacts.


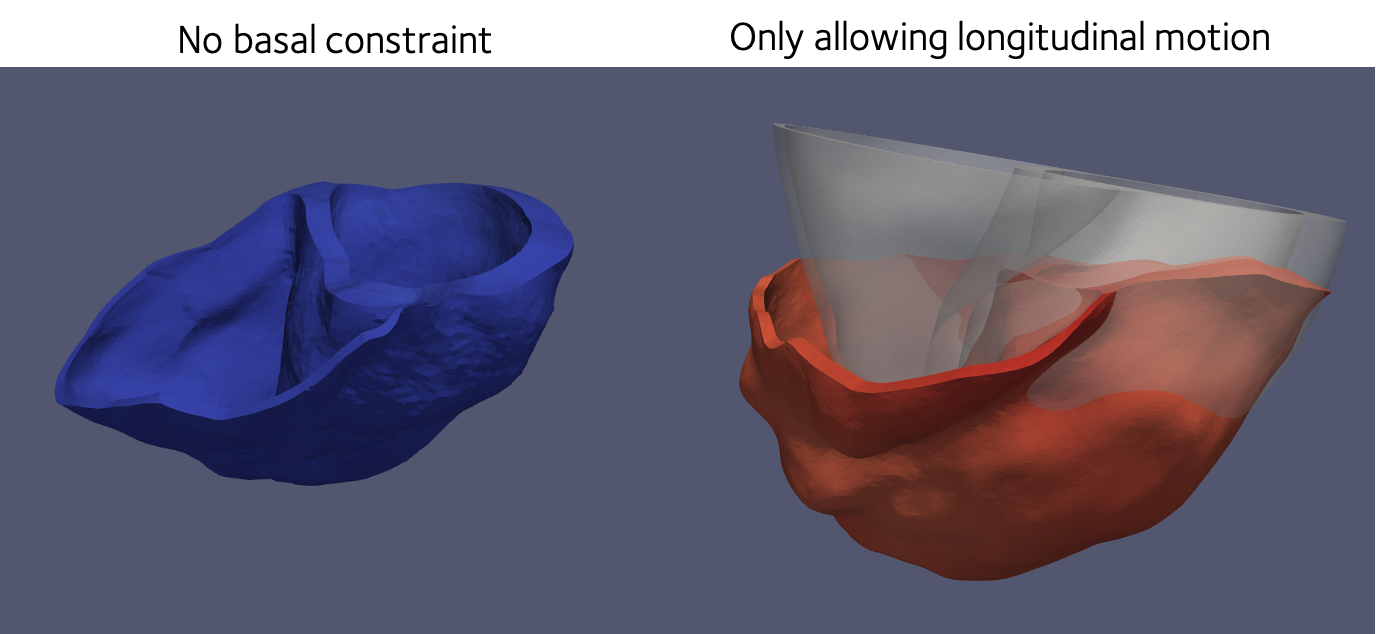


Figure SM10: Demonstrating the effect of allowing the basal plane to move freely (left) or only move in the apex-to-base/longitudinal direction (right).

# Supplementary References

1. Levrero-Florencio F, Margara F, Zacur E, Bueno-Orovio A, Wang ZJ, Santiago A, et al. Sensitivity analysis of a strongly-coupled human-based electromechanical cardiac model: Effect of mechanical parameters on physiologically relevant biomarkers. Comput Methods Appl Mech Eng. 2020 Apr;361:112762.

2. Land S, Park-Holohan SJ, Smith NP, dos Remedios CG, Kentish JC, Niederer SA. A model of cardiac contraction based on novel measurements of tension development in human cardiomyocytes. J Mol Cell Cardiol. 2017;

3. Usyk TP, Mazhari R, McCulloch AD. Effect of laminar orthotropic myofiber architecture on regional stress and strain in the canine left ventricle. J Elast. 2000;61(1–3):143–64.

4. Schoenberg M. Geometrical factors influencing muscle force development. II. Radial forces. Biophys J. 1980 Apr 1;30(1):69–77.

5. Mincholé A, Zacur E, Ariga R, Grau V, Rodriguez B. MRI-Based Computational Torso/Biventricular Multiscale Models to Investigate the Impact of Anatomical Variability on the ECG QRS Complex. Front Physiol. 2019 Aug 27;10:1103.

6. Boukens BJ, Sulkin MS, Gloschat CR, Ng FS, Vigmond EJ, Efimov IR. Transmural APD gradient synchronizes repolarization in the human left ventricular wall. Cardiovasc Res. 2015 May 21;108(1):188–96.

7. Okada J ichi, Washio T, Maehara A, Momomura S ichi, Sugiura S, Hisada T. Transmural and apicobasal gradients in repolarization contribute to T-wave genesis in human surface ECG. Am J Physiol - Hear Circ Physiol. 2011 Jul;301(1):H200–8.

8. Taggart P, Sutton PM, Opthof T, Coronel R, Trimlett R, Pugsley W, et al. Inhomogeneous transmural conduction during early ischaemia in patients with coronary artery disease. J Mol Cell Cardiol. 2000;32(4):621–30.

9. Drouin E, Charpentier F, Gauthier C, Laurent K, Le Marec H. Electrophysiologic characteristics of cells spanning the left ventricular wall of human heart: Evidence for presence of M cells. J Am Coll Cardiol. 1995 Jul 1;26(1):185–92.

10. O’Hara T, Virág L, Varró A, Rudy Y. Simulation of the undiseased human cardiac ventricular action potential: Model formulation and experimental validation. PLoS Comput Biol. 2011 May;7(5).

11. Gima K, Rudy Y. Ionic current basis of electrocardiographic waveforms: A model study. Circ Res. 2002 May 3;90(8):889–96.

12. Plonsey R, Barr RC. Bioelectricity: A quantitative approach. Bioelectricity: A Quantitative Approach. Springer US; 2007. 1–528 p.

13. RAMANATHAN C, RUDY Y. Electrocardiographic Imaging: I. Effect of Torso Inhomogeneities on Body Surface Electrocardiographic Potentials. J Cardiovasc Electrophysiol. 2001 Feb 1;12(2):229–40.

14. Tomek J, Bueno-Orovio A, Passini E, Zhou X, Minchole A, Britton O, et al. Development, calibration, and validation of a novel human ventricular myocyte model in health, disease, and drug block. Elife. 2019 Dec 24;8.

15. Cardone-Noott L, Bueno-Orovio A, Mincholé A, Zemzemi N, Rodriguez B. Human ventricular activation sequence and the simulation of the electrocardiographic QRS complex and its variability in healthy and intraventricular block conditions. 2016 Dec 1;18:iv4–15.

16. Carlsson MB, Trägårdh E, Engblom H, Hedström E, Wagner G, Pahlm O, et al. Left ventricular mass by 12-lead electrocardiogram in healthy subjects: Comparison to cardiac magnetic resonance imaging. J Electrocardiol. 2006 Jan 1;39(1):67–72.

17. Johnson JN, Ackerman MJ. QTc: How long is too long? Vol. 43, British Journal of Sports Medicine. NIH Public Access; 2009. p. 657–62.

18. Feher J. The Heart as a Pump. In: Quantitative Human Physiology. Elsevier; 2012. p. 446–54.

19. Maceira AM, Prasad SK, Khan M, Pennell DJ. Normalized left ventricular systolic and diastolic function by steady state free precession cardiovascular magnetic resonance. J Cardiovasc Magn Reson. 2006;8(3):417–26.

20. Reyhan M, Wang Z, Li M, Kim HJ, Gupta H, Lloyd SG, et al. Left ventricular twist and shear in patients with primary mitral regurgitation. J Magn Reson Imaging. 2015 Aug 1;42(2):400–6.

21. Maceira AM, Prasad SK, Khan M, Pennell DJ. Reference right ventricular systolic and diastolic function normalized to age, gender and body surface area from steady-state free precession cardiovascular magnetic resonance. Eur Heart J. 2006 Dec 1;27(23):2879–88.

22. Garg P, Crandon S, Swoboda PP, Fent GJ, Foley JRJ, Chew PG, et al. Left ventricular blood flow kinetic energy after myocardial infarction - Insights from 4D flow cardiovascular magnetic resonance. J Cardiovasc Magn Reson. 2018 Aug 30;20(1).

23. Nemerovski M, Shah PK, Pichler M, Berman DS, Shellock F, Swan HJC. Radionuclide assessment of sequential changes in left and right ventricular function following first acute transmural myocardial infarction. Am Heart J. 1982 Oct 1;104(4 PART 1):709–17.

24. Bishop A, White P, Oldershaw P, Chaturvedi R, Brookes C, Redington A. Clinical application of the conductance catheter technique in the adult human right ventricle. Int J Cardiol. 1997 Feb 1;58(3):211–21.

25. Zhong X, Spottiswoode BS, Meyer CH, Kramer CM, Epstein FH. Imaging three-dimensional myocardial mechanics using navigator-gated volumetric spiral cine DENSE MRI. Magn Reson Med. 2010 Oct 1;64(4):1089–97.

26. Ponnaluri AVS, Verzhbinsky IA, Eldredge JD, Garfinkel A, Ennis DB, Perotti LE. Model of Left Ventricular Contraction: Validation Criteria and Boundary Conditions. In: Lecture Notes in Computer Science (including subseries Lecture Notes in Artificial Intelligence and Lecture Notes in Bioinformatics). Springer Verlag; 2019. p. 294–303.

27. Bousseljot R, Kreiseler D, Schnabel A. Nutzung der EKG-Signaldatenbank CARDIODAT der PTB über das Internet. Biomed Tech. 1995 Jan 1;40(s1):317–8.

28. Goldberger AL, Amaral LA, Glass L, Hausdorff JM, Ivanov PC, Mark RG, et al. PhysioBank, PhysioToolkit, and PhysioNet: components of a new research resource for complex physiologic signals. Circulation. 2000 Jun 13;101(23).
